# How AI Can Advance Mathematical Biology: Opportunities, Challenges, and Future Directions

**DOI:** 10.1007/s11538-026-01744-x

**Published:** 2026-09-03

**Authors:** Kobra Rabiei, Grzegorz A. Rempala, Reinhard Laubenbacher, Wenrui Hao

**Affiliations:** 1 Department of Mathematics, Penn State University, State College, PA USA; 2 College of Public Health, The Ohio State University, Columbus, OH USA; 3 Department of Medicine, University of Florida, Gainesville, FL USA

**Keywords:** Artificial intelligence, Mathematical biology, Mechanistic models

## Abstract

Mathematical biology has long relied on mechanistic models, including ordinary and partial differential equations, stochastic systems, and agent-based models, to study biological processes across scales. These approaches remain central because they provide structure, interpretability, and biological insight. However, modern biological and biomedical data, including longitudinal clinical records, medical imaging, and multi-omics measurements, are often high-dimensional, noisy, heterogeneous, and incomplete. These features make model calibration, simulation, and uncertainty quantification increasingly difficult. As a result, artificial intelligence (AI) and machine learning (ML) are playing a growing role in mathematical biology, not as replacements for mechanistic modeling, but as complementary tools that can support prediction, hybrid modeling, inference, and control. In this review, we organize these uses of AI from a mathematical biology perspective and examine how data-driven and mechanistic approaches interact across different modeling tasks. The selected examples span biomedical, epidemiological, ecological, evolutionary, biochemical, and population-level systems, while the review is intended to be representative rather than exhaustive. We emphasize recurring challenges that strongly affect biological credibility and practical usefulness, including interpretability, identifiability, generalization, and uncertainty quantification. Our goal is to clarify the roles AI can play in mathematical biology and to highlight the opportunities and limitations that arise when flexible learning methods are integrated with biologically grounded modeling.

## Introduction

The rapid expansion of biological data has changed both the scope and the demands of modern mathematical biology. Longitudinal clinical records, medical imaging, and multi-omics measurements now provide much richer views of biological systems, but they also make modeling, inference, and validation more difficult because observations are often noisy, incomplete, heterogeneous, and only indirectly related to the underlying biological dynamics (Hasin et al. 2017; Qiao et al. 2025; Balsa-Canto et al. 2025). These challenges are especially important in a field that has traditionally relied on structured dynamical models to connect biological mechanisms with data, including ordinary and partial differential equations, stochastic systems, agent-based models, and optimal control frameworks (Cristini et al. 2003; Gumel et al. 2021; Gaff and Lenhart 2009; Laubenbacher et al. 2022; Byrne 2010). Such models remain essential because they provide interpretability and mechanistic insight, but in practice they are often difficult to calibrate, expensive to simulate, and sensitive to identifiability and uncertainty (Raue et al. 2009; Villaverde et al. 2019; Banga and Villaverde 2025; Linden-Santangeli et al. 2025). Recent work on mechanistic medical digital twins further highlights both the promise of personalized computational modeling and the substantial challenges involved in making such models actionable in practice (Laubenbacher et al. 2024).

These limitations have helped drive the growing use of artificial intelligence (AI) and machine learning (ML) in mathematical biology. In this setting, AI is increasingly used not as a replacement for mechanistic modeling, but as a complementary set of tools for prediction, hybrid or gray-box modeling, inference, and control (Yazdani et al. 2020; Lagergren et al. 2020; Daryakenari et al. 2024; Zheng et al. 2022; Sha et al. 2024; Park et al. 2023; Böttcher et al. 2025; Rabiei et al. 2025). Rather than treating mechanistic and data-driven approaches as competing paradigms, recent work increasingly views them as tools that can be used separately or combined depending on the biological question, the available data, and the level of prior knowledge (Liu et al. 2022; Noordijk et al. 2024; Puniya 2025; Qiao et al. 2025). An early example is the hybrid framework of Hamilton et al., which combined mechanistic and data-driven representations to improve dynamical-system prediction (Hamilton et al. 2017).

Despite this progress, the literature remains scattered in disease areas, data modalities, and methodological communities. As a result, methods built on similar computational ideas are often discussed separately even when they play closely related roles relative to a mechanistic model. In this review, we adopt a role-based perspective and focus on four broad uses of artificial intelligence (AI) in mathematical biology: pure data-driven prediction, hybrid or gray-box modeling, inference, and control. Across these areas, we emphasize recurring issues that strongly affect biological credibility and practical usefulness, including interpretability, identifiability, and generalization. Because many biological systems are intrinsically stochastic, these challenges are often intertwined with uncertainty quantification, partial observability, and likelihood-free inference, particularly in biochemical, epidemiological, and population-level settings. The balance of applications differs across the four roles considered here. Section 2 draws mainly on clinical, imaging, and omics studies, where black-box models are widely used for prediction. The gray-box, inference, and control sections cover a broader range of mathematical-biology problems, including differential-equation models, stochastic and agent-based systems, epidemiology, ecology and evolutionary biology, population genetics, biochemical networks, and intervention design. Our aim is therefore to organize representative studies by the role AI plays in modeling, rather than to survey every area of mathematical biology.

**Fig. 1:**
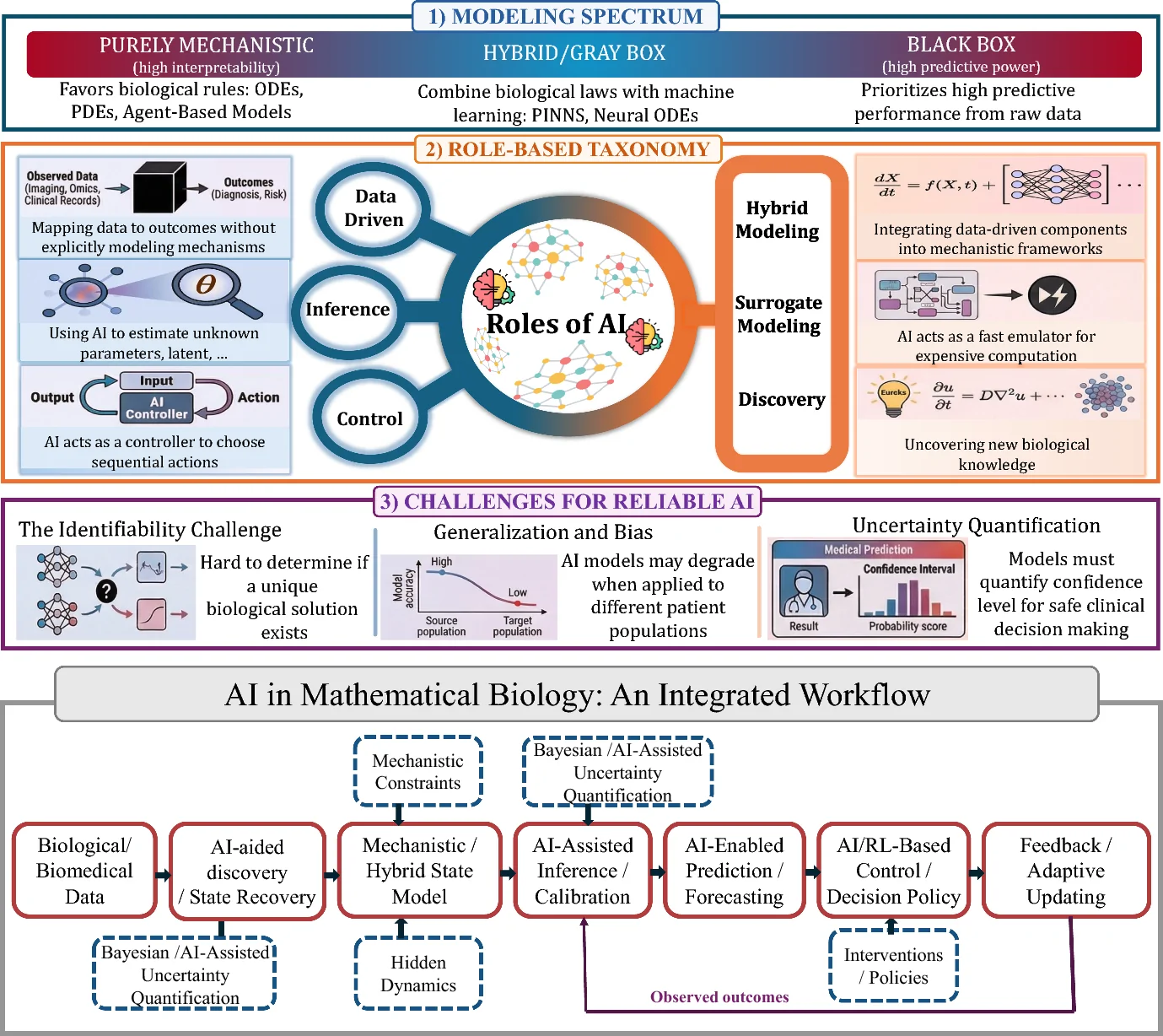
Overview of the modeling spectrum, role-based taxonomy, and integrated workflow of AI in mathematical biology. Top: Three complementary components are shown: (1) a modeling spectrum from purely mechanistic to black-box approaches; (2) a role-based taxonomy comprising data-driven prediction, inference, control, and gray-box modeling, including hybrid, surrogate, and discovery-oriented approaches; and (3) cross-cutting challenges involving identifiability, generalization and bias, and uncertainty quantification. Bottom: An integrated workflow showing how biological data, modeling, inference, prediction, control, and feedback interact. Solid boxes represent the main stages, while dashed boxes denote supporting components. The return arrow indicates that observed outcomes inform subsequent inference and calibration (Color figure online)

Figure 1 summarizes this perspective through two complementary panels. The top panel separates three components: a modeling spectrum ranging from purely mechanistic to black-box approaches; the role-based taxonomy used to organize the review, comprising data-driven prediction, inference, control, and gray-box modeling; and cross-cutting challenges, including identifiability, generalization and bias, and uncertainty quantification. The bottom panel presents a complementary unifying workflow showing how biological data, AI-aided discovery, mechanistic state modeling, inference, prediction, control, and feedback can interact within an integrated AI-enabled mathematical-biology pipeline. Solid boxes represent the primary stages of the workflow, while dashed boxes denote auxiliary modeling components, including mechanistic constraints, hidden dynamics, uncertainty quantification, and intervention policies, that influence multiple stages of the pipeline. The return arrow from *Feedback / Adaptive Updating* to *AI-Assisted Inference / Calibration* indicates that observed outcomes are used to update model states, parameters, or uncertainty before subsequent prediction, control, and adaptive decision-making. Together, the two panels emphasize that AI methods in mathematical biology should be understood both by the roles they play and by how they connect data, models, mechanisms, uncertainty, and interventions. Our goal is not only to summarize representative studies, but also to clarify how these different uses of AI relate to one another and what remains necessary for building methods that are both flexible and biologically grounded.

To clarify the distinction among these roles, Table 1 compares their primary goals, common methodological forms, relationship to mechanistic modeling, typical outputs, and main risks.

**Table 1:** Comparison of the major functional roles of AI in mathematical biology

AI role	Primary purpose	Typical methods	Mechanistic content	Typical output and main risk
Prediction	Diagnosis, prognosis, classification, risk estimation, or forecasting from observed data	Classical ML, CNNs, recurrent networks, transformers, multimodal deep learning	Usually low or indirect; mechanisms are not explicitly represented	Outputs include labels, risk scores, forecasts, subgroups, or response categories. The main risk is that high predictive accuracy may not imply mechanistic insight, causal validity, interpretability, or robustness under dataset shift
Hybrid or gray-box modeling	Complete, accelerate, or approximate mechanistic models using learned components	PINNs, universal differential equations, neural ODEs, neural surrogates, operator learning, symbolic regression	Medium to high; equations, biological constraints, simulators, or mechanistic priors are retained	Outputs include learned dynamical systems, unknown terms, reduced models, surrogate simulators, or discovered mechanisms. The main risk is mechanistic ambiguity, non-identifiability, or poor extrapolation
Inference	Recover parameters, latent states, hidden dynamics, or plausible mechanisms from data	Simulation-based inference, Bayesian inference, neural posterior estimation, variational methods, latent dynamical models	Usually high; inference is performed relative to a generative, statistical, or dynamical model	Outputs include posterior distributions, parameter estimates, latent trajectories, hidden states, or inferred regulatory structures. The main risk is that good data fit may coexist with non-identifiability, weak observability, or large uncertainty
Control and decision-making	Choose interventions, treatment schedules, or policies under evolving biological conditions	Reinforcement learning, model predictive control, neural optimal control, dynamic treatment regimes, causal policy learning	Variable; policies may be learned inside mechanistic simulators or directly from observational trajectories	Outputs include dose schedules, intervention policies, adaptive treatment rules, or feedback controllers. The main risk is failure under model mismatch, simulator artifacts, safety constraints, or clinical distribution shift

The rest of the paper is organized as follows. Section 2 reviews pure data-driven prediction models. Section 3 discusses hybrid or gray-box approaches, including surrogate and closely related discovery-oriented formulations when they remain tied to mechanistic modeling. Section 4 examines inference-focused methods. Section 5 addresses control and decision-making applications. Sections 6 and 7 discuss cross-cutting challenges and future directions, and Sect. 8 offers some conclusions.

## Prediction

One major role of AI in mathematical biology is prediction. In this setting, AI/ML models take observed biological or clinical data as input and predict an output of interest, such as diagnosis, risk stratification, prognosis, treatment response, molecular activity, or future disease trend. The main goal is not to explain the full biological mechanism that generated the data, but to learn a useful input–output relationship from complex measurements. This role is especially useful when the data are high-dimensional, noisy, or difficult to interpret directly, and when accurate prediction is the immediate objective. At the same time, strong predictive performance does not by itself guarantee biological interpretability, robustness, or transportability.

A useful example of AI as a prediction tool is the scFAN framework developed by Fu et al. for single-cell gene regulation (Fu et al. 2020). The biological problem is that transcription-factor binding is important for understanding gene regulation, cell identity, and cellular heterogeneity, but it is difficult to measure directly across the whole genome in individual cells. Thus, the goal is to predict a biologically important quantity that is hard to observe directly.

Fu et al. addressed this challenge by training a deep learning model to predict transcription-factor binding from chromatin-accessibility and sequence-based information. The model is trained using population-level chromatin-accessibility data, DNA sequence information, and genome mappability information, with transcription-factor binding labels obtained from ChIP-seq data. After training, the model is applied to single-cell chromatin-accessibility data, or single-cell ATAC-seq, to predict transcription-factor binding in individual cells. Because single-cell ATAC-seq data are sparse and noisy, the method also aggregates information from similar cells to improve the input signal while retaining cell-level specificity. The predicted binding profiles are then summarized as transcription-factor activity scores, which help characterize cell identity and heterogeneity.

This example illustrates the prediction role of AI because the model learns a direct mapping from biological data to a meaningful biological output. It does not build an explicit dynamical model of gene regulation or infer parameters in a system of equations. Its strength is that it can extract useful regulatory information from sparse single-cell data. Its limitation is that the predictions still require biological validation and do not by themselves explain the causal dynamics of gene regulation. The same prediction role appears in several related settings. In the subsections below, we organize these models according to the type of data and application: structured clinical and biomarker data, time-series and physiological or omics data, medical imaging, and multimodal prediction.

### Structured Clinical and Biomarker Prediction

Classical machine learning methods remain widely used when the data are structured rather than image-based or strongly sequential. Clinical variables, biomarker panels, laboratory measurements, and engineered omics-derived features are often modeled using logistic regression, support vector machines, random forests, and gradient boosting. These methods remain attractive because they are efficient, comparatively easy to implement, and often easier to interpret than deeper architectures.

Such models continue to perform well in biologically relevant settings where the available representation is already informative. Logistic regression has been used successfully in ovarian cancer risk stratification (Van Calster et al. 2020), while ensemble methods have been applied to problems ranging from nanozyme property assessment to mortality prediction in sepsis-related ARDS (Xuan et al. 2025; Xu et al. 2025). In more quantitatively oriented work, data-driven predictors have also been used for the prediction of toxicity, the assessment of protein interactions, and drug screening using engineered molecular descriptors (Wu and Wei 2018; Wang et al. 2020; Feng et al. 2023). Related ideas appear in structured clinical screening tasks, where tree-based learning can provide accurate prediction from relatively small sets of questionnaire or biometric inputs (Ha et al. 2023; Cawiding et al. 2025). More recently, tabular foundation models have introduced a different learning paradigm for structured data. In contrast to logistic regression, random forests, and gradient-boosting methods, which are generally fitted separately to each new dataset, models such as TabPFN are pretrained across large collections of synthetic tabular tasks and can perform prediction on a previously unseen dataset through in-context learning, without conventional task-specific parameter training (Hollmann et al. 2025). This approach is particularly relevant to clinical and biomarker applications, where datasets are often relatively small and may not support the training of highly flexible models from scratch. A more recent example is TabFM, which similarly frames tabular classification and regression as a zero-shot in-context learning problem and is designed to operate across larger and more heterogeneous tables (Kong and Das 2026). These models may reduce the need for repeated model fitting, feature engineering, and extensive hyperparameter optimization. However, their use in mathematical biology still requires careful assessment of whether the pretraining distribution adequately represents the biological data of interest, together with evaluation of calibration, interpretability, robustness, and external validity.

### Time-Series and Physiological or Omics Prediction

Deep learning has become increasingly influential in prediction tasks built around temporally ordered, sequential, or otherwise high-dimensional biological data. Many biological and clinical processes unfold over time or across latent developmental trajectories, and their measurements are naturally represented as sequences or structured profiles, including longitudinal records, physiological signals, and single-cell omics data. Recurrent, convolutional, and attention-based architectures are well-suited to such settings because they can model complex dependencies directly.

Beyond clinical time-series, related ideas extend to molecular and cellular prediction tasks. The scFAN example above belongs to this group because it uses deep learning to predict transcription-factor binding at single-cell resolution from chromatin-accessibility data (Fu et al. 2020). Machine learning has also been used to distinguish anti-PD-1 responders from non-responders using single-cell transcriptomic data (Liu et al. 2022). These studies show how pure prediction models can remain useful in mathematically oriented biological research when the objective is classification, response prediction, or feature extraction rather than explicit dynamical interpretation. Reviews of influenza forecasting and epidemic early warning systems similarly highlight the importance of temporal and sequence-aware prediction in public-health settings (Adeoye et al. 2025; El Morr et al. 2024).

### Medical Imaging Prediction

Medical imaging is one of the most prominent application areas for deep learning in predictive modeling. Images such as CT, MRI, ultrasound, and histopathology slides contain high-dimensional spatial information that is difficult to capture with handcrafted features alone, so convolutional neural networks and related architectures are now widely used for diagnosis, classification, and prognosis (Suzuki 2017; Zhou et al. 2021).

In oncology, deep learning has been used for tumor detection, classification, and outcome prediction from radiological and histopathological data (Jiang et al. 2023; Litjens et al. 2017). These models can capture morphological and textural patterns that are difficult to encode through conventional pipelines, and they have increasingly been linked to survival, recurrence, and treatment response (Mobadersany et al. 2018; Yuan et al. 2025). Similar ideas appear in neurology and liver cancer, where imaging-based AI supports diagnosis, grading, and prognostic assessment (Abdusalomov et al. 2023; Noori Mirtaheri et al. 2025; Pellat 2023; Martinino et al. 2022). Although much of this literature comes from adjacent biomedical AI communities rather than mathematical biology in the narrow sense, it remains relevant here because imaging has become an important quantitative source of predictive biological information.

### Multimodal Prediction

Many biological and clinical questions cannot be addressed adequately through a single modality. Relevant information is often distributed across imaging, clinical variables, omics measurements, laboratory data, and other contextual sources. Multimodal prediction models aim to integrate these complementary inputs to improve predictive performance and provide a broader representation of the system under study.

This has become an especially active area of deep learning because heterogeneous inputs can be combined within a shared predictive framework. In breast cancer, for example, multimodal deep learning has been used to integrate FDG PET-CT with multi-omics information for long-term prognostication (Liang et al. 2026). Similar approaches have been reported in cervical cancer prognostic modeling under radiotherapy (Wang et al. 2025), in cancer prognosis systems that combine pathology-style representations with structured clinical information (Hou et al. 2026), and in broader multi-omics prediction settings (Reel et al. 2021; Acharya and Mukhopadhyay 2024).

Overall, pure prediction models remain attractive because of their practical flexibility and empirical performance. However, their scientific role is narrower than that of mechanistic or hybrid approaches: they are most effective when the goal is accurate classification, risk prediction, or forecasting from observed data, rather than mechanistic explanation or causal understanding. Table 2 summarizes representative prediction studies discussed in this section.

**Table 2:** Representative AI/ML-based pure prediction studies in mathematical biology and adjacent quantitative biomedicine

References	Domain	Data type	Model type	Contribution
Van Calster et al. (2020)	Ovarian cancer	Clinical variables + biomarkers	Logistic regression	Validated an interpretable clinical model with strong discrimination for benign versus malignant adnexal masses and meaningful subtype stratification
Wu and Wei (2018)	Toxicology	Molecular descriptors / chemical structure	Multitask deep neural network	Used topology-based representations and multitask deep learning for quantitative toxicity prediction across multiple toxicity endpoints
Wang et al. (2020)	Protein engineering / molecular biophysics	Protein structural descriptors + mutation features	Topology-based ML / network tree	Predicted mutation-induced changes in protein–protein binding affinity using data-driven topology-informed learning
Feng et al. (2023)	Drug repurposing	DrugBank molecular and binding descriptors	Machine learning screening	Applied machine learning to prioritize existing compounds for opioid use disorder based on predicted molecular interactions
Ha et al. (2023)	Sleep disorder screening	Questionnaire-based clinical variables	Machine learning classifier	Showed that a simple questionnaire coupled with machine learning can accurately predict sleep disorder risk in a screening setting
Cawiding et al. (2025)	Sleep disorder prediction	Body composition / biometric variables	Tree-based machine learning	Developed a simplified prediction framework using body composition metrics for accurate sleep disorder prediction
Fu et al. (2020)	Single-cell regulatory genomics	Single-cell chromatin accessibility and sequence-derived features	Deep learning	Predicted transcription factor binding at single-cell resolution from high-dimensional regulatory genomics data
Liu et al. (2022)	Cancer immunotherapy response	Single-cell transcriptomics	Machine learning	Distinguished anti-PD-1 responders from non-responders and identified resistance-associated cellular patterns from transcriptomic data
Yuan et al. (2025)	Pan-cancer pathology	Whole-slide histopathology	Weakly supervised deep learning	Demonstrated large-scale pathology-based outcome prediction across multiple cancer cohorts using whole-slide images
Liang et al. (2026)	Breast cancer	PET-CT + multi-omics	Multimodal deep learning	Showed that integrating imaging and omics improves long-term prognostication in breast cancer
Wang et al. (2025)	Cervical cancer	Clinical + imaging data	Multimodal deep learning	Illustrated multi-center prognostic modeling using multimodal deep learning in a radiotherapy setting

## Hybrid AI Modeling (Gray-Box)

A second major role of AI in mathematical biology is hybrid or gray-box modeling. This role occupies the space between purely mechanistic modeling and purely data-driven prediction. In this setting, the biological system is not treated as fully unknown, but it is also not assumed to be completely specified. Instead, mechanistic structure is retained where biological knowledge is available, while AI is used to represent unresolved processes, accelerate expensive simulators, or help recover missing structure from data. This role is especially important in mathematical biology, where ordinary differential equations (ODEs), partial differential equations (PDEs), stochastic systems, and agent-based models (ABMs) often capture essential biology but may remain incomplete, computationally expensive, or difficult to calibrate under sparse and noisy observations (Liu et al. 2022; Noordijk et al. 2024; Puniya 2025; Gherman et al. 2023; Norton et al. 2026).

In this review, we use the term gray-box modeling for approaches that retain an explicit connection to mechanistic knowledge while introducing a learned component. This connection can take three main forms. First, in structure-preserving hybrid models, known equations, constraints, or biological relationships are built directly into the learned model, while unknown terms are estimated from data. Second, simulator emulators are trained on outputs from a mechanistic model to provide a faster approximation; they inherit the scientific assumptions of the original simulator but do not alter its governing structure. Third, mechanism-discovery methods use learning to recover candidate equations, interactions, or functional forms that can be interpreted and tested within a mechanistic framework. Graph-based methods may fit within the first or third category, depending on whether biological structure is built into the graph or learned from data. These distinctions organize the subsections that follow and clarify that mechanistic content may enter through the model structure, the source of the training data, or the form of the learned output.

A useful example of the gray-box role is the biologically informed neural network framework developed by Lagergren et al. for modeling collective cell migration from sparse experimental data (Lagergren et al. 2020). The biological problem comes from scratch-assay experiments, where a layer of cells is scratched and the cell population gradually migrates back into the empty region. These experiments are commonly used to study wound healing and cancer cell migration, and they are naturally modeled using reaction–diffusion equations for cell density. However, even when the general PDE structure is known, the exact forms of the diffusion and growth terms are often unclear.

### Equation-Constrained and Partially Learned Dynamical Models

One of the most visible developments in gray-box modeling has been the rise of equation-constrained learning. A widely used framework in this area is the physics-informed neural network (PINN), which embeds governing differential equations directly into the training objective so that the model is trained not only to match observations but also to satisfy the underlying system equations (Raissi et al. 2019). In biological applications, this PINN-style idea has motivated biology-informed variants, including systems-biology-informed neural-network formulations for ODE-based systems biology problems, where mechanistic constraints are used to infer hidden dynamics, unobserved species, and unknown parameters from limited measurements (Yazdani et al. 2020). Biologically informed neural networks further extend this equation-constrained perspective to reaction–diffusion PDE models by retaining broad PDE structure while learning unresolved nonlinear diffusion and reaction terms directly from sparse biological data (Lagergren et al. 2020).

The effectiveness of such equation-constrained neural approaches also depends strongly on how derivatives are computed during training, and recent work has shown that automatic differentiation is not merely a technical convenience but a central ingredient in successfully training neural networks for differential-equation problems (Chen et al. 2025). This equation-constrained structure is particularly attractive when data are sparse or noisy, since the mechanistic structure acts as a regularizer and restricts predictions to biologically plausible regimes. More recently, gray-box systems-biology frameworks have combined PINNs with symbolic regression so that parameter estimation and missing-mechanism identification can be carried out within a single interpretable pipeline (Daryakenari et al. 2024).

A complementary family of methods is built around neural differential equations. Universal differential equations provide a general formulation in which part of the right-hand side of a dynamical system is replaced by a learnable function while the broader mechanistic scaffold is preserved (El-Gazzar and Gerven 2025). This is especially useful when a model is believed to be structurally meaningful but incomplete in detail. Biochemically informed neural ODEs further extend this framework by preserving the stoichiometric structure of biochemical systems while using neural networks to represent unknown process rates and incorporate prior knowledge through process-specific inputs and qualitative constraints (Fonseca et al. 2026). Related neural ODE frameworks have been used in neurodegeneration to learn patient-specific dynamics for Alzheimer’s disease (Jung et al. 2026) and in cardiovascular modeling to replace difficult physiological terms while preserving global circulation structure (Grigorian et al. 2024). More broadly, causality-aware and mechanistically constrained dynamical formulations have also begun to introduce stronger structural restrictions to improve interpretability and distinguish direct regulation from indirect effects in intervention-sensitive settings (Park et al. 2023).

These approaches now appear across several biological domains. In epidemiology, physics-informed deep learning has been used to combine compartmental disease models with neural representations of external covariates for structured infectious disease forecasting (Qian et al. 2025). In oncology, related ideas have been explored through digital-twin-style reconstruction of patient-specific prostate cancer growth trajectories (Camacho-Gomez et al. 2025). In a related but more discovery-oriented direction, data-driven causal model discovery has been used to build personalized dynamical representations of Alzheimer’s disease progression while retaining an explicitly mechanistic causal interpretation (Zheng et al. 2022). In cancer mathematical biology, data-driven ODE formulations have also been used to connect immune profiling with osteosarcoma progression while preserving a mechanistic dynamical view of tumor–immune interactions (Le et al. 2021). Taken together, these studies illustrate a core strength of gray-box modeling: machine learning is used not simply to predict outputs, but to complete, personalize, or constrain a mechanistic system.

### Surrogate and Reduced Mechanistic Models

Another important role of gray-box AI is surrogate modeling. Rather than learning an unknown term inside the governing equations, surrogate models emulate the behavior of an existing mechanistic simulator with a cheaper approximation. In mathematical biology, the simulator may take the form of an ODE, PDE, stochastic biochemical model, or ABM. The scientific assumptions remain those of the original simulator, but machine learning is used to make repeated forward evaluation, calibration, optimization, sensitivity analysis, or uncertainty quantification more scalable (Gherman et al. 2023; Norton et al. 2026).

Neural networks are among the most widely used surrogates because they can approximate nonlinear mappings from parameters, initial conditions, or controls to simulator output. In deterministic settings, they have been used to emulate compartmental epidemic models such as SIR- and SEIR-type systems, enabling much faster forward evaluation and more practical parameter estimation (Kurul et al. 2025). In developmental biology, U-Net-style architectures have been used to emulate spatial cell-based simulations of vasculogenesis and recover relevant spatial patterns at a fraction of the original computational cost (Comlekoglu et al. 2025). Operator- and closure-learning approaches extend this idea beyond standard vector-to-vector approximation toward coarse-grained or field-level emulation in spatially distributed systems. Recent neural-operator work shows that nonlinear reaction–diffusion dynamics can be learned efficiently through Laplacian eigenfunction-based representations, further strengthening the role of surrogate learning for spatially distributed mechanistic systems (Wang and Hao 2025). For example, data-driven methods have been used to learn black-box and gray-box chemotactic PDE closures agent-based Monte Carlo simulations, thereby linking fine-scale stochastic dynamics to interpretable macroscopic equations (Lee et al. 2023). These examples show how surrogate modeling can accelerate repeated mechanistic analysis while remaining tied to the original biological simulator.

Agent-based models are especially natural targets for surrogates because they are often expensive and frequently used in many-query settings. Reviews of surrogate-assisted ABM analysis have highlighted their roles in calibration, sensitivity analysis, and uncertainty quantification across a wide range of biological applications (Norton et al. 2026). Epidemiological studies have developed surrogate methods for complex individual-based models, allowing rapid approximation of stochastic epidemic outputs without repeated high-cost simulation (Davis et al. 2020). Related work has also connected high-dimensional agent-based models to multidimensional data through surrogate modeling, allowing computationally expensive ABM behavior to be explored more efficiently across parameter space (Bergman et al. 2024). Neural surrogates can also make posterior estimation and inverse modeling more practical in ABM calibration (Anirudh et al. 2022). In this sense, surrogates are not merely computational conveniences; in many settings they are what make repeated mechanistic analysis practically feasible.

In some applications, the surrogate is valuable not only because it is faster, but because it preserves enough structure to support additional scientific tasks. Differentiable surrogate models can provide gradients cheaply, making PDE-constrained inverse problems and parameter estimation much more tractable than repeated direct simulation (Zhang et al. 2026). Other surrogate strategies aim to remain more interpretable by learning reduced mechanistic descriptions of more detailed stochastic or agent-based systems. Equation-learning methods can recover interpretable differential-equation surrogates directly from stochastic ABM simulations, providing a general framework for translating agent-level dynamics into lower-dimensional population models (Nardini et al. 2021). In cancer modeling, such surrogates have been used to study equilibrium behavior and treatment-response relationships without repeatedly simulating the full stochastic system (Burrage et al. 2025). Related work has shown that lower-dimensional surrogate models can support intervention design and control while maintaining a clear mechanistic connection to the original simulator (Fonseca et al. 2025). More generally, model-reduction approaches have argued that simplified phenomenological descriptions can still remain meaningfully tied to the underlying mechanistic parameters rather than simply replacing them (Transtrum and Qiu 2016). Surrogate modeling is also relevant in stochastic biochemical systems, where the object of interest may be an evolving probability distribution rather than a single trajectory. Neural approximations to the Chemical Master Equation, for example, act as surrogates for molecule-number distributions as functions of time and parameters, enabling faster exploration of stochastic reaction systems (Sukys et al. 2022).

Multiscale modeling provides another natural setting in which gray-box and surrogate approaches can strengthen mathematical biology. Many biological systems involve interacting processes across molecular, cellular, tissue, and population scales, so useful models often require some form of scale bridging, coarse-graining, or reduced-order approximation. Classical multiscale theory for stochastic reaction networks provides a mathematical foundation for reducing complex systems while preserving key dynamical behavior (Ball et al. 2006; Kang et al. 2019; Kim et al. 2017). AI-assisted surrogates and learned reduced models can build on this foundation by approximating expensive multiscale simulators, learning effective coarse-scale dynamics, or propagating uncertainty across scales. In this role, machine learning is most useful when it accelerates or complements mechanistic scale reduction rather than replacing the biological interpretation of the original multiscale model.

### Mechanistically Guided Discovery

Gray-box modeling also includes a discovery-oriented role when AI is used to reveal previously unknown structures while remaining tied to explicit dynamics or mechanistic constraints. In such cases, the output is not merely a prediction or a fast emulator, but a candidate mechanistic completion: a missing term, a compact reduced law, a causal interaction, or a regulatory structure that can be interpreted biologically.

One major direction is governing-equation and dynamical-law discovery. In many biological settings, the relevant ODE or PDE model is only partially known, or several competing mechanistic formulations remain plausible. Equation-learning methods have been adapted to practical limitations of biological data. Nardini et al. developed methods for learning dynamical equations when only a small number of temporal samples are available (Nardini et al. 2020), while Lagergren et al. addressed the recovery of biological transport PDEs from noisy spatiotemporal observations (Lagergren et al. 2020). Equation learning has also been used to derive interpretable differential-equation models from stochastic ABM simulations. These learned equations can remain accurate in parameter regimes where standard mean-field approximations are inadequate (Nardini et al. 2021).

Related discovery-oriented approaches have also been applied in ecology and evolutionary biology. Bonnaffé et al. used neural ordinary differential equations to learn nonparametric functional forms governing population dynamics from time-series data. Applied to hare–lynx population data, the method inferred intraspecific density dependence and interspecific predator–prey interactions and identified a central equilibrium point underlying the observed oscillations (Bonnaffé et al. 2021). Machine learning can also support the interpretation of already specified ecological models. Simon et al. applied random forests to simulations from an ontogenetically stage-structured consumer–resource model and used feature and interaction analyses to reduce complex stability patterns to three core ecological mechanisms linking plant demography and trophic allocation. This illustrates an interpretive use of AI in which learning does not replace the mechanistic simulator but helps identify interpretable drivers of its behavior (Simon et al. 2023).

More broadly, symbolic and hybrid equation-learning methods can recover candidate dynamical structures while preserving mathematical interpretability (Massonis et al. 2023; Wu et al. 2025). Mechanism-discovery approaches have also been used to infer biologically meaningful functional forms directly from sparse experimental observations, including cell-invasion models with uncertainty quantification (VandenHeuvel et al. 2022). Bayesian formulations further emphasize that uncertainty in the discovered dynamical structure should itself be quantified, particularly when data are limited and several compact models remain plausible (Martina-Perez et al. 2021). The resulting models remain mathematically explicit and can therefore be examined as candidate biological mechanisms rather than only as black-box predictors. An emerging extension of equation discovery is to pretrain models across broad collections of dynamical systems and then apply them to previously unseen trajectories. ODEFormer provides an early example of this direction. It is a transformer model pretrained on synthetic ODE systems to infer multidimensional dynamical equations in symbolic form from a single observed trajectory (Ascoli et al. 2024). Unlike conventional symbolic regression methods that begin the discovery process separately for each dataset, this approach transfers information learned across many dynamical systems to a new inference problem. This connects foundation-model pretraining with the mechanism-discovery role of gray-box modeling.

A second direction is causal and regulatory discovery under dynamical or mechanistic constraints. Here, the role of AI is to narrow the space of plausible biological mechanisms while preserving explicit structural assumptions. Model-based causal inference methods have been developed to distinguish direct regulation from synchrony and indirect effects by testing whether time-series data are reproducible under general monotonic ODE systems (Park et al. 2023). Related computational frameworks have identified translated biochemical reaction networks with special topological structure, enabling analytical characterization of their steady states and stochastic long-term behavior (Hong et al. 2023). Mechanistically informed regulatory-network discovery also fits naturally within this direction when the goal is to recover interpretable biological structure while remaining tied to explicit system dynamics (Hossain et al. 2024; Brown et al. 2025; Chevalley et al. 2025). Similarly, retrieval-augmented generation has been used to extract Alzheimer’s disease biomarker relations from the literature and build candidate causal networks, extending discovery-oriented modeling beyond directly data-fitted dynamical systems (Zhou et al. 2025). In this context, AI contributes not only as a fitting tool but also as a means of constraining and refining biologically plausible mechanisms.

A broader mechanism-discovery role appears when hybrid frameworks are used to extract latent biological programs, interpretable developmental structure, or candidate decision rules from complex data. Some studies are best understood in this way when their main contribution is biological insight rather than improved simulation alone (Camacho-Gomez et al. 2023; Myers et al. 2021). For this reason, we treat discovery as part of gray-box modeling rather than as a fully separate category: the structures being discovered typically emerge from learning procedures that remain anchored to mechanistic representations, and their scientific value depends on preserving that connection.

### Graph- and Network-Based AI for Structured Biological Systems

Many biological systems are not naturally represented as independent feature vectors, but as networks of interacting units. Examples include epidemic contact networks, signaling pathways, gene-regulatory networks, cell–cell communication graphs, spatial tissue neighborhoods, and higher-order interaction systems. Graph- and network-based AI methods are therefore especially relevant to mathematical biology because they provide a way to combine relational biological structure with flexible learning. In this setting, nodes may represent individuals, cells, genes, proteins, tissue regions, or compartments, while edges encode contacts, regulatory interactions, spatial adjacency, or mechanistic coupling. Graph neural networks and message-passing architectures extend deep learning beyond Euclidean data by allowing information to propagate along network structure (Bronstein et al. 2017; Hamilton et al. 2017). However, a graph neural network is not inherently mechanistic or gray-box. In this review, we consider a graph-based model to be gray-box only when its nodes, edges, message-passing rules, conservation constraints, or update functions retain explicit biological or mechanistic meaning. For example, an epidemic model may inherit its edges from a contact network, so transmission is restricted to biologically possible pathways. Similarly, a signaling or gene-regulatory model may use a known interaction network while learning uncertain interaction strengths or dynamics. A graph may also be inherited from a mechanistic simulator, with the learned model preserving its compartmental structure or conservation rules. By contrast, a graph learned only to improve predictive performance, without interpretable structure or update rules, is better viewed as a black-box model. Network-based epidemic models illustrate how biologically meaningful graph structure can connect individual-level contacts to population-level dynamics (Kiss et al. 2026).

At the same time, graph-based AI introduces familiar mathematical challenges in a new form. Learned edges, message-passing weights, or inferred network structures may be difficult to interpret mechanistically, and different networks can sometimes generate similar aggregate behavior. As a result, graph-based learning in mathematical biology should be coupled with identifiability analysis, uncertainty quantification, and biological validation when the goal is not only prediction but mechanistic explanation or intervention design.

### Data-Side Support for Reliable AI-Enabled Modeling

The reliability of gray-box modeling also depends on the representations and labels constructed before model fitting begins. Biological data, particularly single-cell measurements, are often sparse, noisy, and sensitive to preprocessing choices. Consequently, manually selected thresholds, distorted representations, or unstable cluster labels can introduce hidden bias that is subsequently inherited by prediction, inference, mechanism-discovery, and control methods. This data-side role is complementary to the structure-preserving gray-box methods discussed above. Rather than learning an unknown component inside a mechanistic model, it uses statistically grounded data-driven procedures to construct more reliable inputs for downstream modeling.

The scLENS framework illustrates this role in single-cell RNA-sequencing analysis. It combines an additional normalization step with random-matrix-theory-based noise filtering and a signal-robustness test to distinguish biological signal from noise without requiring the user to select the signal dimension manually (Kim et al. 2024). A related concern arises after dimensionality reduction, because stochastic clustering algorithms can produce different labels across repeated runs. The scICE framework addresses this problem by evaluating the consistency of cluster labels across stochastic clustering trials and identifying a smaller set of reliable clustering solutions (Kim et al. 2025). These examples show that uncertainty and reproducibility should be considered not only after a model has been fitted, but also when determining which signals, representations, and labels are supplied to the model. Although these methods do not themselves embed a mechanistic equation, they support the interface between raw biological data and subsequent mechanistic or gray-box analysis. Table 3 summarizes representative gray-box studies together with selected data-side methods that support the reliability of downstream AI-enabled and mechanistic modeling.

**Table 3:** Representative gray-box and supporting data-driven studies in mathematical biology

References	Biological setting	Modeling base	Data-driven component	Scientific role
Raissi et al. (2019)	General dynamical systems	ODE/PDE constraints	PINN	Equation-constrained learning framework for forward and inverse problems
Yazdani et al. (2020)	Systems biology	ODE model	Deep learning with ODE constraints	Hidden dynamics and parameter inference within a mechanistic system
Lagergren et al. (2020)	Wound healing/cell biology	Reaction–diffusion PDE	Biologically informed neural network	Learning unknown mechanistic terms from sparse biological data
Daryakenari et al. (2024)	Systems biology	Dynamical system model	PINN + symbolic regression	Gray-box identification and missing-mechanism discovery
El-Gazzar and Gerven (2025)	General dynamical systems	ODE model	Universal differential equation	Embedding learnable terms inside mechanistic dynamics
Grigorian et al. (2024)	Cardiovascular modeling	Lumped ODE system	Hybrid neural ODE	Replacing difficult physiological terms while preserving global structure
Park et al. (2023)	Regulatory dynamics	Monotonic ODE system	Model-based causal inference	Mechanistically constrained discovery of direct regulatory interactions
Kurul et al. (2025)	Epidemiology	ODE epidemic simulator	Neural surrogate	Fast emulator for repeated simulation and calibration
Comlekoglu et al. (2025)	Developmental biology	Spatial cell-based simulator	U-Net surrogate	Spatial emulator for expensive biological simulations
Lee et al. (2023)	Chemotaxis / multiscale biology	ABM-to-PDE closure	Black-box / gray-box closure learning	Learning interpretable chemotactic PDE closures from agent-based simulations
Davis et al. (2020)	Epidemiology	Individual-based epidemic model	Mixture density network	Stochastic surrogate for repeated epidemic simulation
Kim et al. (2024, 2025)	Single-cell transcriptomics	Statistical structure of noisy scRNA-seq data	Noise filtering and clustering-consistency analysis	Reliable signal extraction and stable label construction before downstream modeling.
Burrage et al. (2025)	Cancer dynamics	ABM	Equation-learning surrogate	Reduced the mechanistic ODE surrogate from a stochastic simulator
Fonseca et al. (2025)	Biomedical ABMs / digital twins	ABM	Reduced mechanistic surrogate	Surrogate-enabled control while preserving mechanistic linkage
Sukys et al. (2022)	Systems biology	CME / stochastic reaction network	Neural distribution surrogate	Distribution-level approximation for stochastic biochemical models
Massonis et al. (2023)	Biological dynamical systems	ODE model discovery	Sparse / interpretable model discovery	Recovery of identifiable and interpretable dynamical models from biological data
VandenHeuvel et al. (2022)	Cell invasion	PDE-based invasion model	Mechanism discovery with uncertainty quantification	Discovery of biologically meaningful functional forms from sparse experimental observations
Hong et al. (2023)	Biochemical reaction networks	Reaction-network dynamics	Computational translation framework	Discovery of special network structure and long-term behavior

## Inference

A third major role of AI in mathematical biology is inference. In this setting, AI/ML methods are used to estimate hidden quantities from observed data, such as unknown parameters, latent variables, hidden states, unobserved species, regulatory interactions, or uncertainty in model predictions. This role is distinct from prediction, where the main goal is to map inputs to outputs, and from gray-box modeling, where AI is often used to complete, modify, or discover components of the mechanistic model itself. In inference, the central question is instead: given a biological model or generative process, what hidden quantities could have produced the observed data? Thus, while gray-box modeling uses AI to shape the model structure, inference typically starts from a specified model and uses AI to recover latent quantities, estimate uncertainty, and assess consistency with observed data. The relationship between inference and gray-box modeling can therefore be iterative. Inference diagnostics may indicate that an assumed simulator should be revised rather than only recalibrated. A practical workflow is to first use posterior predictive checks, systematic residual patterns, lack-of-fit tests, and sensitivity analyses to determine whether disagreement with the data is primarily caused by parameter uncertainty or structural model inadequacy. When no plausible parameter values allow the simulator to reproduce the observed behavior, allowing fitted parameters or latent states to absorb the discrepancy can produce misleading biological interpretations. In such cases, the gray-box approaches reviewed in Sect. 3 provide a principled next step: learned discrepancy terms can represent unresolved processes, partially specified differential equations can recover missing dynamics, and mechanism-discovery methods can propose candidate structural revisions. The revised model should then be reassessed using the same inference diagnostics, creating an iterative cycle of diagnosis, model refinement, and validation.

An example of AI as an inference tool comes from a cell-migration problem closely related to the gray-box example discussed above (Lagergren et al. 2020), but with a different scientific goal. Kimpson, Flegg, and Simpson study collective cell migration in scratch and barrier assays, a setting relevant to wound healing, tissue development, and cancer invasion (Kimpson et al. 2026). However, in this case, the aim is not to learn missing terms in a governing equation or to refine the model structure. Instead, the stochastic agent-based random-walk model is already specified, and AI is used to infer its unknown parameters from observed spatial data.

In these models, individual cells move, divide, and may show directional bias. The unknown parameters describe biologically meaningful processes such as cell motility, initial cell density, directional bias, and proliferation rate. The main difficulty is that these stochastic agent-based models usually do not have a tractable likelihood function, so standard likelihood-based methods such as maximum likelihood estimation or Markov chain Monte Carlo cannot be applied directly. Approximate Bayesian computation is possible, but it can be computationally expensive and sensitive to tolerance choices and summary statistics. Replacing the stochastic simulator with a deterministic PDE surrogate is another option, but this can introduce bias and requires an additional noise model.

The authors addressed this problem using neural posterior estimation. They simulated the stochastic cell-migration model under many parameter values and trained a neural density estimator to learn the relationship between simulated observations and the parameters that generated them. After training, the method can be applied to observed migration data to approximate the posterior distribution of the unknown parameters. They demonstrated this framework on a hierarchy of random-walk models, including baseline migration, directional bias, proliferation, and a combined model with both bias and proliferation. They also compared inference from one-dimensional column-count summaries with inference directly from two-dimensional spatial data using a convolutional neural network. This comparison also highlights the difference between dimensionality reduction and inference-preserving representation learning. A compact summary or neural embedding may describe or reconstruct the observations well without retaining the features needed to identify model parameters or distinguish competing biological mechanisms. Its inferential adequacy can be evaluated through parameter-recovery tests on held-out simulations and by comparing inference results obtained from the full observations, handcrafted summaries, and learned embeddings. Robustness can be assessed by varying noise levels, sampling density, or image resolution, while nuisance invariance requires testing whether the representation is insensitive to variation unrelated to the parameters of interest. Model-discrimination experiments can further determine whether the representation preserves features that separate competing mechanistic explanations. For spatial biological data, topological and other structure-aware summaries may be useful because they retain information about connectivity, clustering, shape, and spatial organization that generic low-dimensional embeddings may discard.

This example illustrates the inference role of AI because the neural network is used to recover hidden parameters and quantify uncertainty for an existing stochastic biological model. The biological simulator remains the assumed generative model, while AI makes Bayesian parameter inference feasible when the likelihood is intractable. Thus, the example is closely related biologically to the gray-box example, but it serves a different methodological purpose: it shows AI as a tool for parameter inference rather than model completion.

AI-based inference appears in several related settings. In the subsections below, we organize these approaches into simulation-based and likelihood-free inference, physics-informed and mechanistic inference, neural differential equations and latent dynamics, and approximate Bayesian inference with uncertainty quantification.

### Simulation-Based and Likelihood-Free Inference

Simulation-based inference (SBI) has become one of the central paradigms for AI-driven inference in mathematical biology. In many biological systems, especially stochastic and agent-based models, the likelihood function is unavailable or prohibitively expensive to compute. SBI replaces explicit likelihood evaluation with simulation and learns the relationship between parameters and observations from synthetic data. Foundational work has clarified how neural density estimation can support likelihood-free posterior inference in simulator-based models (Cranmer et al. 2020; Durkan et al. 2018).

Stochastic mechanistic systems are especially important in this context because randomness is often part of the biological mechanism rather than merely observational noise. Stochastic reaction networks, branching processes, continuous-time Markov chains, and stochastic epidemic models can represent intrinsic demographic variability, molecular fluctuations, random contact structure, and partially observed event dynamics (Wilkinson 2009; Kang and Kurtz 2013; Rempała 2023). For such systems, the likelihood may be analytically unavailable or computationally expensive, and the observed data may be only a partial or noisy projection of the underlying process. This makes stochastic simulation-based inference particularly relevant: AI-based density estimators, latent representations, and amortized inference methods can help connect stochastic mechanistic simulators to observed biological data while retaining an explicit generative interpretation.

A particularly influential family of methods is neural posterior estimation (NPE), in which deep density estimators, often based on normalizing flows, are trained to approximate posterior distributions directly from simulations. These methods can reduce the need for hand-crafted summary statistics and support amortized inference, making posterior estimation rapid for new observations once training is complete. In the biological literature, NPE has been applied in spatiotemporal stochastic models of cell migration and mechanobiology (Kimpson et al. 2026; Arruda et al. 2026), as well as in epidemiological and phylodynamic settings (Voznica et al. 2022). In population genetics, Min et al. trained neural posterior estimators on simulated genetic data to approximate posterior distributions for evolutionary and demographic parameters. They evaluated the approach using both summary statistics and raw genotype data and applied it to infer historical effective population sizes and the demographic history of *Drosophila melanogaster* (Min et al. 2026). Together, these studies show how likelihood-free Bayesian inference can scale to problems where mechanistic simulators are complex but still informative.

SBI is also becoming increasingly important when observations are spatially structured or high-dimensional. Representation-learning approaches have used convolutional autoencoders to embed simulations and observations in a shared latent space, enabling likelihood-free calibration of agent-based models from high-dimensional imaging data (Wang et al. 2026). These studies show that SBI can remain effective even when the observations are experimentally rich and the likelihood is inaccessible. Topological data analysis (TDA) provides a complementary approach for constructing informative summary statistics from complex spatial and spatiotemporal model outputs. Rather than relying only on learned latent representations, TDA captures persistent geometric and connectivity features of spatial patterns. Such descriptors have been used to distinguish parameter regimes in a spatial angiogenesis model (Nardini et al. 2021) and to quantify collective migration patterns in cell-based agent-based models (Nguyen et al. 2024). More recently, TDA-based summaries have been incorporated into multi-objective Bayesian inference and rule selection for an agent-based model of zebrafish pattern formation (Liu and Volkening 2026). Together, these studies illustrate a progression from spatial-pattern characterization to parameter and model inference. Despite these advantages, SBI remains conditional on the assumed simulator and on the design of the simulations used for training. If the simulator omits important biological processes, the observed data fall outside the simulated distribution, or the prior and proposal distributions do not adequately cover the relevant parameter space, the resulting posterior may be poorly calibrated or misleading. Reliability should therefore be examined using posterior predictive checks, simulation-based calibration, and coverage diagnostics (Talts et al. 2018). Sensitivity to prior and proposal choices should also be assessed, together with direct comparisons between simulated and observed data. Benchmark-based comparisons across tasks and performance metrics can provide additional evidence about inference quality (Lueckmann et al. 2021), while explicit assessment of model discrepancy is needed when the simulator may be misspecified (Huang et al. 2023). These checks can help distinguish uncertainty in the parameters from a more fundamental discrepancy between the simulator and the biological system.

### Physics-Informed and Mechanistic Inference

A second major direction in AI-based inference is the integration of machine learning with mechanistic models, particularly through PINNs and related equation-constrained approaches. These methods embed known biological laws, often in the form of ordinary or partial differential equations, into neural architectures, allowing parameters or latent states to be inferred while preserving mechanistic structure.

Foundational work on PINNs established the general framework for solving inverse problems under differential-equation constraints (Raissi et al. 2019). In biological applications, related approaches have been used to infer time-varying parameters and hidden trajectories in compartmental epidemic models (Ning et al. 2023). More recent work in computational biology has also shown that hybrid neural ODE frameworks can support parameter estimation while addressing identifiability and robustness, helping inference remain biologically interpretable rather than acting as unconstrained function fitting (Giampiccolo et al. 2024; Yazdani et al. 2020).

Beyond the estimation of scalar or time-varying parameters, equation-constrained learning can also be used to infer an entire unknown function embedded within a governing equation. This functional-inference setting lies near the boundary between parameter inference and gray-box modeling: the broader mechanistic structure is specified, but an unresolved functional component is recovered from data. For example, Density-PINNs infer the distribution of signal-transduction times directly from single-cell response-time traces, allowing features of the underlying signaling pathway to be investigated without prescribing the distribution in advance (Jo et al. 2024). In another application, IC-PINN infers coupling functions between interacting oscillators from sparse and noisy time-series observations without requiring a predetermined basis representation (Hwang et al. 2026). These examples extend mechanistic inverse problems from finite-dimensional parameter estimation to functional inference. However, the identifiability, regularization, uncertainty, and biological interpretation of the learned function remain important considerations.

This mechanistic perspective also extends to data collection and model calibration. Bayesian frameworks have been used to fit dynamical models while guiding data collection through optimal experimental design, as in ODE-based models of haematopoiesis (Lomeli et al. 2021). Together, these studies illustrate why equation-constrained inference is appealing in mathematical biology: estimation is required to remain compatible with known system dynamics and, in some settings, with the experimental design process itself.

### Neural Differential Equations, Latent Dynamics, and Mechanistic Structure Inference

Neural differential equations provide another important framework for inference. In these models, unknown parts of the dynamics may be learned while known mechanistic structure is retained, allowing data-driven flexibility to be incorporated without discarding the broader system formulation. Related biologically informed NeuralODE frameworks have been developed for gene regulatory dynamics, where prior biological structure is used to improve interpretability and realism (Hossain et al. 2024). Closely related mechanistically informed inference methods have also been used to recover delayed regulatory effects in partially observed gene regulation processes (Hong et al. 2023), while executable mechanistic models of gene expression have been used to combine network inference and simulation within a single framework (Ventre et al. 2023).

This line of work becomes especially relevant in regulatory-network and latent-structure inference. Optimization-based approaches have been developed to infer cell lineages and intercellular communication networks from single-cell transcriptomic data (Wang et al. 2019), while deep learning and optimal transport have been used to reconstruct growth and temporal trajectories from single-cell transcriptomic snapshots (Sha et al. 2024). These inference problems are also naturally connected to graph-based AI, since regulatory interactions, cell–cell communication, and spatial tissue organization can often be represented as networks whose structure must be inferred, constrained, or updated from data (Bronstein et al. 2017; Hamilton et al. 2017).

More broadly, AI has also enabled latent dynamical and generative inference methods that aim to recover hidden states and continuous-time evolution when the underlying dynamics are only indirectly observed. Latent force models provide one route for combining mechanistic structure with Gaussian-process flexibility (Álvarez et al. 2011), while structured latent-dynamical formulations remain useful for inferring nonlinear hidden dynamics in biological systems, including stochastic epidemic processes with partially observed dynamics (Xu et al. 2016). In single-cell biology and related areas, neural ODEs, variational autoencoders, and optimal-transport-inspired approaches are increasingly used to reconstruct latent biological structure and continuous trajectories from snapshot observations (Cao et al. 2022). Reviews of generative models for cell dynamics show that this is becoming a major direction in biological inference, especially when direct temporal measurements are unavailable (Richter et al. 2026).

Taken together, these studies show that neural differential-equation and latent-dynamical inference place learning inside a structured dynamical system rather than around it, supporting interpretable continuous-time representations of hidden biological processes.

### Approximate Bayesian Inference and Uncertainty Quantification

Approximate Bayesian inference remains an important foundation for mathematical biology. Methods such as approximate Bayesian computation, Bayesian synthetic likelihood, integrated nested Laplace approximations, and variational inference provide scalable alternatives when exact Bayesian inference is impractical. Recent review work in epidemiology highlights both the progress and the trade-offs of these approaches, especially in settings where computational efficiency must be balanced against inferential fidelity (Li et al. 2025).

This computational bottleneck is distinct from the likelihood-free setting discussed in Sect. 4.1. Even when a likelihood can be evaluated, conventional Markov chain Monte Carlo methods may become prohibitively expensive in high-dimensional mechanistic models because they require many repeated model evaluations and may mix slowly. Variational inference instead approximates the posterior through optimization and can therefore provide a faster alternative, although standard formulations may be sensitive to constrained parameter boundaries and local optima. Annealed and transformed variational inference addresses these difficulties by combining a flexible deep-generative variational distribution with a surjective transformation that reduces posterior truncation near parameter boundaries and a temperature-annealing procedure that guides the optimization through a sequence of intermediate posterior distributions (Cho et al. 2025). Applications to infectious-disease compartmental models illustrate how this approach can accelerate calibration while retaining a close approximation to more computationally intensive Bayesian methods. This line of work therefore provides a bridge between the neural density-estimation methods used in simulation-based inference and scalable likelihood-based calibration. Nevertheless, the accuracy and calibration of the resulting posterior approximation must still be evaluated rather than inferred from computational speed alone. Inference quality, however, depends on more than posterior estimation alone. Identifiability and observability are central mathematical issues because they determine whether biologically meaningful quantities can be recovered from the available measurements. Structural identifiability asks whether parameters are uniquely determined in principle under ideal noise-free observations, whereas practical identifiability asks whether they can be estimated reliably from finite, noisy, and often partially observed data (Raue et al. 2009; Simpson et al. 2020; Wang and Hao 2025). Observability is closely related but focuses on whether hidden states, unknown inputs, and parameters can be reconstructed from measured outputs of a dynamical system (Villaverde et al. 2019). These questions are especially important in biological models because different parameter combinations, latent trajectories, or competing mechanisms may generate nearly indistinguishable observations.

The problem becomes sharper when AI components are embedded inside mechanistic models. Flexible learned terms can improve data fit, but they may also introduce additional non-identifiability or mechanistic ambiguity if multiple learned corrections explain the same residual structure. In this setting, uncertainty quantification should not be viewed only as a confidence interval around fitted parameters; it should also be used to assess parameter sloppiness, sensitivity geometry, observability of latent states, and distinguishability among competing mechanistic explanations (Raue et al. 2009; Villaverde et al. 2019; Simpson et al. 2020; Wang and Hao 2025). This is especially important in digital-twin settings, where identifiability-guided assessment can help determine which personalized parameters and trajectories can be interpreted reliably from clinical data (Jiang et al. 2025). A model may fit the data well yet still fail to support reliable mechanistic conclusions, so uncertainty and identifiability are not auxiliary diagnostics but part of the inference problem itself.

Recent work also shows how uncertainty-aware inference can be adapted to specific mechanistic settings. Survival dynamical systems translate population-level epidemic ODE models into individual-level survival and hazard functions for outbreak-data analysis (KhudaBukhsh et al. 2020). Bayesian filtering and forecasting approaches have also been used in clinical infectious-disease settings, including short-term hospital influenza prediction with Kalman-filter-based models (Mani et al. 2026). Likelihood-based logistic-regression approaches have further been proposed for structure and parameter inference in stochastic reaction networks, including partially observed epidemic settings (Choi et al. 2025). Taken together, these studies show that uncertainty-aware inference in mathematical biology includes not only general Bayesian machinery, but also model-specific inferential frameworks tied closely to biological dynamics. Table 4 summarizes representative inference studies across likelihood-free, mechanistic, and latent-dynamical settings.

**Table 4:** Representative AI/ML-based inference studies in mathematical biology

References	Domain	Model type	Inference type	Contribution
Voznica et al. (2022)	Epidemiology / phylodynamics	Epidemic or phylodynamic simulator	Neural posterior estimation	Posterior inference in complex disease systems using simulation-based inference
Min et al. (2026)	Population genetics	Population-genetic simulator	Neural posterior estimation	Demographic and evolutionary parameter inference
Kimpson et al. (2026)	Cell migration / wound healing	Stochastic agent-based random-walk model	Neural posterior estimation	Likelihood-free Bayesian parameter inference for motility, directional bias, proliferation, and initial cell density from spatial cell-migration data
Wang et al. (2026)	Cancer / spatial imaging	ABM + convolutional autoencoder	Representation-based likelihood-free inference	Calibration of spatial agent-based models from high-dimensional imaging data
Raissi et al. (2019)	General	Differential-equation model + PINN	Inverse / mechanistic inference	Foundational PINN framework for inverse problems under differential-equation constraints
Ning et al. (2023)	Epidemiology	Compartmental epidemic model + PINN	Mechanistic state and parameter inference	Inference of hidden trajectories and time-varying parameters in epidemic systems
Giampiccolo et al. (2024)	Computational biology	Hybrid neural ODE	Mechanistic parameter inference	Robust parameter estimation with identifiability analysis in biological dynamical systems
Lomeli et al. (2021)	Hematopoiesis	ODE model + Bayesian design	Mechanistic inference / experimental design	Dynamical-model calibration linked to optimal experimental design
Hossain et al. (2024)	Systems biology	Biologically informed NeuralODE	Mechanistic dynamical inference	Structured inference for gene regulatory dynamics
Hong et al. (2023)	Gene regulation	Mechanistic dynamical model	Latent regulatory inference	Inference of delayed effects in partially observed gene regulation processes
Ventre et al. (2023)	Systems biology	Mechanistic gene-expression model	Mechanistic network inference	Combines gene regulatory network inference and simulation within one mechanistic framework
Wang et al. (2019)	Single-cell systems biology	Optimization-based dynamical model	Lineage / communication inference	Cell-lineage and intercellular communication-network inference from single-cell data
Sha et al. (2024)	Single-cell systems biology	Optimal transport + deep learning	Trajectory inference	Reconstruction of growth and temporal trajectories from snapshot single-cell data
Álvarez et al. (2011)	Latent dynamical systems	Latent force model + Gaussian process	Probabilistic latent-dynamics inference	Hidden continuous-time dynamics with uncertainty quantification
Xu et al. (2016)	Epidemic dynamics	Structured latent-dynamical model	Latent-state inference	Inference of partially observed nonlinear epidemic dynamics
Cao et al. (2022)	Single-cell biology	Variational autoencoder + optimal transport	Generative latent-structure inference	Reconstruction of latent biological structure from high-dimensional single-cell observations

## Control and Decision-Making

A fourth major role of AI in mathematical biology is control and decision-making. In this setting, the goal is not only to predict how a biological system may evolve, but also to choose interventions that can steer the system toward a desired outcome. These interventions may include drug dosing, treatment sequencing, stimulation protocols, or public-health policies. This role has deep roots in mathematical biology: before recent AI-based approaches, optimal control theory was already widely used to study vaccination strategies, treatment scheduling, and therapeutic intervention design through differential-equation models and Pontryagin-type optimality systems (Lenhart and Workman 2007; Neilan and Lenhart 2010; Siewe and Friedman 2022, 2023). Recent AI-based approaches should therefore be viewed not as replacements for classical control theory, but as extensions that support adaptive, data-driven, and feedback-based decision-making in more complex settings.

The methods grouped under control and decision-making differ in the model information they require, the data from which decisions are learned, and the form of the resulting policy. Classical model-based optimal control assumes a specified dynamical system and computes an intervention that minimizes a prescribed objective over a fixed horizon; in biological applications, the controls are often continuous dose or intervention-intensity functions. Model predictive control also uses a predictive model, but repeatedly solves a shorter-horizon optimization problem as new state measurements become available, thereby producing feedback. Reinforcement learning instead learns a policy or value function from interactions with a simulator or environment, or from previously collected transitions in the offline setting. It may be model-based or model-free and can accommodate either discrete or continuous actions. Dynamic treatment regimes define sequences of treatment rules tailored to a patient’s evolving history and are commonly estimated from longitudinal randomized or observational data; the available treatments are often discrete or clinically constrained, although continuous dosing rules are also possible. Causal policy learning explicitly targets the counterfactual value of alternative policies and therefore requires identification assumptions such as consistency, positivity, and sequential exchangeability, particularly when observational data are used. These categories are not mutually exclusive: model-based reinforcement learning may use a mechanistic simulator, and causal reinforcement learning may be used to estimate a dynamic treatment regime (Aiello et al. 2024; Laber et al. 2014; Zhang and Bareinboim 2020; Gottesman et al. 2019).

A useful example of AI as a control tool is the work on controlling medical digital twins with artificial neural networks (Böttcher et al. 2025). The biological motivation is that personalized medicine often requires computational models that can be updated with patient-specific data and used to guide interventions. However, realistic medical digital twins may be multiscale, stochastic, hybrid, or agent-based. Such models are often difficult to control using standard optimal-control methods designed mainly for differential-equation systems.

The authors addressed this challenge by developing neural-network-based controllers for agent-based medical digital twins. In this framework, the neural network represents the control policy: it learns how to choose an intervention based on the current state of the model. The policy is trained using the simulated system dynamics so that the controller can steer the biological model toward a desired target. This is different from using AI only to predict disease progression. Here, AI is part of the decision layer: it selects actions that change the future trajectory of the system.

This example illustrates the control role of AI because the main output is not a diagnosis, a parameter estimate, or a missing model term, but an intervention policy. Its main contribution is to extend policy optimization to complex stochastic and agent-based simulators that do not have a convenient ODE representation for standard optimal-control methods.

AI-based control and decision-making appear in several related settings. In the subsections below, we organize these approaches into treatment optimization and adaptive therapy in oncology, epidemic and population-level intervention control, and physiological feedback control and clinical policy learning.

### Treatment Optimization and Adaptive Therapy in Oncology

Oncology provides one of the clearest examples of AI as a control tool in mathematical biology because cancer treatment is naturally a sequential intervention problem under evolving tumor dynamics. Early studies framed chemotherapy dosing as a reinforcement-learning problem in which the controller adjusts drug administration according to tumor or patient state rather than following a fixed schedule (Padmanabhan et al. 2017; Treesatayapun et al. 2023). These studies recast dosage selection as a closed-loop control problem and showed that even relatively simple reinforcement-learning formulations can outperform rigid protocols when the objective is to balance efficacy and toxicity. Related ideas have also been explored extensively in classical mathematical oncology, where mechanistic dynamical systems have been used to study tumor–immune interactions, adaptive therapy, and threshold-guided intervention strategies without relying on AI-based controllers (Huo et al. 2025; Tang et al. 2025).

More recent work moves beyond fixed chemotherapy schedules toward adaptive therapy and multi-step treatment design. Model-driven deep reinforcement learning has been used to discover personalized adaptive-therapy rules from mathematical tumor models, showing how AI can exploit eco-evolutionary tumor dynamics rather than merely react to the current state (Gallagher et al. 2024). This line of work has also expanded to multiscale treatment-design problems, where reinforcement learning is coupled to mathematical models of tumor evolution and microenvironmental interaction in order to optimize combination-treatment schedules under changing biological conditions (Liu et al. 2025). Sequential drug-design frameworks such as SequenTx push this idea further by coupling reinforcement learning with evolving tumor-response landscapes, so that later treatment choices depend on how earlier therapies reshape tumor vulnerability (Chen et al. 2026). Together, these studies illustrate how AI can be integrated with mathematical models of resistance, adaptation, and state transition to guide dosing, timing, and treatment sequencing decisions.

### Epidemic and Population-Level Intervention Control

A second major theme is epidemic and public-health control, where interventions act on transmission dynamics at the population level rather than on an individual patient trajectory. The clearest mathematically grounded examples place the control directly inside a mechanistic epidemic model and learn the intervention policy end-to-end. In this setting, neural networks are not used only for prediction; they are embedded within the epidemic dynamics to optimize time-dependent interventions, making AI part of the control layer of a differential-equation model rather than a separate black-box decision tool (Yin et al. 2023).

Reinforcement learning has also been used to design epidemic intervention policies when decision-makers must choose among changing response levels over time. In these studies, actions such as tightening or relaxing non-pharmaceutical interventions are selected sequentially based on the epidemic state, and the learned policy is evaluated against outcomes such as infections, deaths, or cumulative cost (Du et al. 2023; Zhang et al. 2026). Some work emphasizes hierarchical decision structures, allowing the controller to switch among qualitatively different intervention modes rather than only adjusting a single continuous control variable (Du et al. 2023). Compared with classical optimal control, these approaches are often better suited to settings with discrete actions, adaptive feedback, or complex policy constraints, although their biological interpretability is usually weaker than in more explicit mechanistic formulations.

This perspective also extends beyond a single well-mixed population. Multi-agent reinforcement learning has been applied to HIV intervention planning across interacting jurisdictions, where each region is treated as a decision-making unit but the epidemiological effects remain coupled across the broader system (Sharma et al. 2025). Taken together, these studies show that AI can contribute to epidemic and public-health control at several levels, from learning intervention functions within mechanistic epidemic models to supporting adaptive policy design across coupled population systems (Yin et al. 2023; Du et al. 2023; Zhang et al. 2026; Sharma et al. 2025).

### Physiological Feedback Control and Clinical Policy Learning

AI-based control is also emerging in medical settings where interventions must be updated continuously from physiological or neural feedback. In diabetes management, one representative study integrates data-driven glucose prediction into a model predictive control framework for the artificial pancreas (Aiello et al. 2024). Here, machine learning does not replace the controller itself; instead, it improves the predictive component used to choose insulin delivery. A related direction appears in adaptive neuromodulation, where reinforcement learning has been used to adjust deep brain stimulation parameters for Parkinson’s disease and tremor suppression in simulated neural environments (Zhao et al. 2025). In both settings, the controller operates in a closed loop, repeatedly updating the intervention on the basis of the evolving physiological or neural state.

Not all AI-based decision-making in this area is built around an explicit mechanistic dynamical model. A related line of work formulates treatment selection as a sequential decision problem learned from clinical trajectories, randomized studies, or observational data. Early empirical work showed that reinforcement learning could support multi-stage clinical decision-making under missingness and complex patient histories (Shortreed et al. 2011). More recent studies have considered offline learning in high-stakes settings such as sepsis, where policies must be estimated retrospectively from ICU data without unrestricted online exploration (Tu et al. 2025).

A more formal framework is provided by dynamic treatment regimes, which define sequences of treatment rules tailored to a patient’s evolving state (Laber et al. 2014; Zhang and Bareinboim 2020). Within this framework, causal reinforcement learning, uncertainty-aware counterfactual inference, and counterfactually guided policy transfer address how treatment rules can be learned under confounding and domain shift (Zhang and Bareinboim 2020; Wu et al. 2024; Killian et al. 2022). Safety-constrained dosage regimes further show how toxicity and adverse-event risk can be incorporated directly into the decision objective rather than treated only as post hoc evaluation criteria (Laber et al. 2018). This literature broadens AI-based control from policies trained within mechanistic simulators to sequential treatment rules learned from longitudinal clinical data, while making causal identification assumptions central to their interpretation.

Across these applications, learned intervention policies require a stronger validation standard than predictive accuracy alone because policy decisions alter the future state of the biological system and may change the distribution of subsequent observations. A useful validation framework has several stages. First, the learned policy should be compared with appropriate clinical and mathematical baselines, such as current practice, fixed or rule-based protocols, and classical optimal-control or model-predictive-control strategies, using the same measures of efficacy, toxicity, constraint violations, and resource use. Second, the policy should be stress-tested under parameter uncertainty, measurement noise, structural model uncertainty, and alternative plausible system dynamics rather than being evaluated only in the simulator used for training. Third, evaluation should include out-of-distribution scenarios involving different initial conditions, disease severities, patient populations, adherence patterns, and rare but clinically important events. Finally, evidence should progress from retrospective or offline evaluation and simulation studies to experimental validation and, where relevant, carefully monitored prospective studies before deployment. High predictive accuracy of a state model or high estimated policy value in simulation does not by itself establish safety, causal validity, robustness, or clinical benefit (Gottesman et al. 2019).

Table 5 summarizes representative studies on AI-based control and decision-making across oncology, epidemic intervention, physiological feedback control, and clinical policy learning.

**Table 5:** Representative papers on the role of AI in control and decision-making for mathematical biology

References	Subsection	Disease / biological system	AI / model type	Contribution
Gallagher et al. (2024)	Treatment optimization and adaptive therapy in oncology	Tumor evolution / adaptive therapy	Deep RL + mathematical tumor model	Strong mathematical-biology example combining mechanistic tumor dynamics with AI-based control for personalized adaptive therapy scheduling
Liu et al. (2025)	Treatment optimization and adaptive therapy in oncology	Multiscale tumor evolution and treatment response	RL + multiscale mathematical modeling	Extends AI-based control from single-scale tumor response to multiscale treatment design under evolving biological conditions
Chen et al. (2026)	Treatment optimization and adaptive therapy in oncology	Evolving tumor landscape	RL + tumour-response framework	Uses evolving treatment response to guide multi-step therapy design rather than one-step dosing
Padmanabhan et al. (2017)	Treatment optimization and adaptive therapy in oncology	Chemotherapy dosing	Reinforcement learning	Early example framing cancer therapy as a closed-loop dosing problem under changing tumor or patient state
Yin et al. (2023)	Epidemic and population-level intervention control	Epidemic ODE systems	Neural-network optimal control / deep learning	Directly links AI-based control with mechanistic epidemic differential-equation models
Zhang et al. (2026)	Epidemic and population-level intervention control	UK COVID-19	RL + calibrated epidemic simulation	Uses a data-calibrated epidemic environment rather than a purely abstract simulator for intervention design
Sharma et al. (2025)	Epidemic and population-level intervention control	HIV population dynamics	Multi-agent RL	Public-health example with interacting decision-makers across a coupled population-level system
Aiello et al. (2024)	Physiological feedback control and clinical policy learning	Diabetes / artificial pancreas	Data-driven predictor + MPC	Representative example of learning-enhanced control embedded within a physiological feedback-control framework
Zhao et al. (2025)	Physiological feedback control and clinical policy learning	Parkinson’s disease / tremor suppression	RL + neural simulation	Closed-loop neuromodulation example in which stimulation actions are adapted to neural-system state
Zhang and Bareinboim (2020)	Physiological feedback control and clinical policy learning	Dynamic treatment regimes	Causal RL	Important bridge between causal inference and sequential decision-making for multi-stage treatment design
Laber et al. (2018)	Physiological feedback control and clinical policy learning	Clinical dosage policy under safety constraints	Dynamic treatment regime / statistical decision framework	Strengthens the safety dimension of policy learning by explicitly incorporating constrained treatment design

## Challenges and Limitations

The methods reviewed in Sects. 2, 3, 4 and 5 can fail in different ways. A prediction model may be accurate without identifying a biological mechanism; a gray-box model may fit the observations while learning an incorrect missing term; an inference method may return a concentrated posterior under a misspecified simulator; and a control policy may perform well in simulation but fail under biological or clinical variability. These problems require different diagnostics, which we organize into five challenges.

### Biological and Causal Validity

The prediction models in Sect. 2 learn associations between clinical, imaging, or omics measurements and an outcome. Feature importance, attention scores, and latent representations may describe which information the predictor uses, but they do not establish that the selected variables cause the biological outcome.

The gray-box models in Sect. 3 retain equations, interaction networks, or mechanistic simulators, but this structure does not automatically validate the learned component. A neural correction may compensate for an incorrect parameter, an omitted state, or measurement bias while still reducing trajectory error (Noordijk et al. 2024). Even when a learned model is embedded in an ODE or biological network, causal interpretation depends on whether the assumed structure and direction of interactions are valid (Park et al. 2023).

The distinction becomes more consequential in control. A treatment policy learned from observational clinical trajectories requires assumptions that permit valid counterfactual comparisons between alternative treatments (Laber et al. 2014; Zhang and Bareinboim 2020). A policy trained in a mechanistic simulator is instead conditional on the treatment-response relationships represented by that simulator and therefore requires independent evaluation before deployment (Gottesman et al. 2019).

### Identifiability, Observability, and Model Discrepancy

The inference problems reviewed in Sect. 4 require more than a good fit or a narrow posterior. Structural identifiability determines whether model parameters can be recovered uniquely in principle, practical identifiability concerns whether they can be estimated reliably from finite and noisy data, and observability determines whether hidden states or unknown inputs can be reconstructed from the available outputs (Raue et al. 2009; Villaverde et al. 2019; Simpson et al. 2020; Wang and Hao 2025).

Gray-box models introduce an additional ambiguity because changes in mechanistic parameters, initial conditions, and learned correction terms may generate nearly identical trajectories. Mechanism-discovery methods may similarly produce several equations or interaction structures that explain the same observations. Without additional experiments, constraints, or identifiability analysis, the learned component cannot be interpreted as a unique missing mechanism.

Parameter uncertainty must also be distinguished from structural model inadequacy. Systematic residual patterns or posterior predictive failure that cannot be removed by plausible parameter changes indicate that the simulator may be missing relevant biology (Huang et al. 2023). Continuing to recalibrate such a model can force parameters or latent states to absorb the discrepancy. In that setting, constrained discrepancy terms, partially learned differential equations, or mechanism-discovery methods provide a more appropriate route for representing and testing the missing dynamics (Daryakenari et al. 2024; Massonis et al. 2023).

### Robustness and Generalization

Generalization has a different meaning for each methodological role. For the prediction models in Sect. 2, a random train–test split does not establish performance across hospitals, scanners, assay platforms, patient populations, or missing-data patterns. External cohorts and acquisition settings are needed to test whether the learned associations remain stable.

For the surrogates and learned dynamical terms in Sect. 3, low error within the training distribution does not establish accuracy outside the parameter, state, or initial-condition ranges used to generate the training data. Mechanistic structure may improve extrapolation, but learned components and surrogates can still fail when the system enters a new dynamical regime (Noordijk et al. 2024). Recent approaches address this limitation by pooling information across multiple experiments and parameter values when learning continuum models from stochastic spatial agent-based simulations (Ciocanel et al. 2026), or by incorporating physical parameters directly into sparse equation discovery to support extrapolation to previously unseen parameter regimes (Nicolaou et al. 2023).

For simulation-based inference in Sect. 4, the observed biological data may lie outside the simulated training distribution or in a poorly sampled region of parameter space. In that case, an amortized inference network may return a confident posterior even though it has not learned the relevant parameter–data relationship.

Control creates a stronger distribution-shift problem because the policy changes the future state distribution. A learned controller may move the system into regions that were rarely represented during training or exploit numerical and structural artifacts in the simulator (Gottesman et al. 2019). Evaluation should therefore include external data, alternative plausible simulators, parameter perturbations, and deliberately constructed out-of-distribution states rather than only held-out samples from the original development distribution.

### Sources and Propagation of Uncertainty

Uncertainty should be distinguished by its source because different sources require different diagnostics. *Observational uncertainty* arises from measurement error, missingness, preprocessing, and finite sampling, whereas *intrinsic stochasticity* is generated by the biological process itself, as in stochastic reaction networks, epidemic models, and agent-based systems (Wilkinson 2009; Kang and Kurtz 2013; Rempała 2023). The former should be represented through an observation model, while the latter must be included in the system dynamics.

*Parameter uncertainty* concerns unknown biological rates or coefficients, and *latent-state uncertainty* concerns unobserved species, trajectories, or patient states. Both are central to the inference methods in Sect. 4 and are closely connected to identifiability and observability (Raue et al. 2009; Villaverde et al. 2019). *Model-selection uncertainty* arises when several equations or networks explain the same observations, while *learned-component uncertainty* concerns neural correction terms, surrogate approximations, or discovered functions that are not uniquely determined by the data (Martina-Perez et al. 2021).

*Structural model discrepancy* is different from parameter uncertainty. It occurs when no plausible parameter values allow the simulator to reproduce the observed data. Posterior predictive checks and comparisons between observed and simulated behavior are therefore needed to detect model inadequacy rather than allowing fitted parameters or latent states to absorb the discrepancy (Huang et al. 2023).

These uncertainty sources affect the four roles differently. Prediction requires calibrated risks under observational and model uncertainty. Gray-box modeling must separate uncertainty in mechanistic parameters from uncertainty in learned terms, competing structures, and simulator discrepancy. Inference requires reliable uncertainty for parameters and latent states, assessed through posterior predictive checks, simulation-based calibration, coverage analysis, and held-out simulations (Talts et al. 2018; Lueckmann et al. 2021).

In control, uncertainty must be propagated to intervention outcomes rather than evaluating a single fitted trajectory. Measurement uncertainty, intrinsic stochasticity, parameter and state uncertainty, and uncertainty in model structure or learned components should be carried into predicted treatment benefit, toxicity, constraint violations, and policy value. A policy that performs well only for one parameter estimate or one simulator realization is not adequately validated (Gottesman et al. 2019).

### Benchmarking, Reproducibility, and Deployment

The benchmark must match the scientific role of the method. Prediction requires external discrimination and calibration. Surrogate modeling requires error assessment throughout the relevant simulator design region. Mechanism discovery requires recovery of interpretable and distinguishable structures. Inference requires coverage, calibration, and identifiability analysis, as emphasized in benchmarking studies of simulation-based inference (Lueckmann et al. 2021). Control requires comparison with current practice, fixed or rule-based protocols, and classical optimal-control or model-predictive-control baselines.

Reproducibility depends on reporting the choices that determine the analysis. For prediction, these include preprocessing, cohort selection, and missing-data handling. For surrogates and simulation-based inference, the simulator equations, parameter ranges, priors, proposal distributions, and simulation design should be reported. For control, the state definition, action space, reward function, safety constraints, stopping rules, and evaluation environment must be specified (Gottesman et al. 2019).

Deployment evidence should increase with the consequence of failure. A prediction model may require external validation, whereas an intervention policy should progress from simulation and retrospective evaluation to experimental and, where appropriate, prospective validation before it is used to guide biological or clinical decisions (Gottesman et al. 2019).

These limitations show why prediction, gray-box modeling, inference, and control cannot be evaluated using a common performance measure. The required evidence depends on whether the claimed output is an association, a learned mechanism, an inferred quantity, or a recommended intervention.

## Future Perspectives

Building on the challenges identified in Sect. 6, the representative studies summarized in Tables 2, 3, 4 and 5 suggest several directions for future work in AI and mathematical biology. These directions do not arise equally from every paper reviewed here. Rather, they reflect recurring opportunities across studies that connect learning with biological structure, dynamical inference, uncertainty quantification, and intervention design.

The first direction is the principled use of foundation models and scientific large language models for knowledge extraction, hypothesis generation, model construction, and scientific-workflow automation.

Their ability to transfer knowledge across tasks and, in some cases, operate on new datasets with limited or no task-specific retraining makes them promising tools for prediction, gray-box modeling, inference, and control (Hollmann et al. 2025; Ascoli et al. 2024). The central opportunity is not only to improve predictive performance or automate individual tasks, but also to develop foundation models that are biologically interpretable, mechanistically informed, and uncertainty-aware. Interpretability, calibration, robustness, and biological validation are not unique requirements for foundation models; they also apply to the convolutional, multimodal, and reinforcement-learning methods discussed earlier in this review.

In the taxonomy used here, foundation models and large language models do not constitute a fifth methodological role. The four roles are defined by the scientific function of the output rather than by the architecture used to produce it. A foundation model used to predict a biological or clinical outcome belongs to prediction; one used to learn or complete a dynamical system belongs to gray-box modeling; one used to estimate parameters or latent states belongs to inference; and one used to construct an intervention policy belongs to control. Large language models and retrieval-augmented systems more often provide enabling infrastructure across these roles by supporting literature synthesis, knowledge extraction, model formulation, code generation, experimental design, and interpretation (Zhou et al. 2025; Newsham et al. 2025). These applications are best viewed as scientific-workflow automation unless the system directly produces one of the four scientific outputs, in which case it should be evaluated according to the corresponding role.

One concrete realization of this direction would be a foundation model for mechanistic structure. Such a model would be pretrained across broad families of dynamical systems and would use this prior knowledge to analyze a new biological system from limited observations. It should go beyond trajectory prediction or recovery of a single equation by accommodating partially observed states, irregular measurements, intrinsic stochasticity, and multiple candidate mechanisms. It should also incorporate biological constraints, quantify uncertainty, and indicate when the observations are insufficient to distinguish among competing explanations. These capabilities could connect pretraining more directly with gray-box modeling, mechanistic discovery, and inference, while making identifiability and observability part of the model output rather than only post hoc diagnostic checks.

A second direction is the development of predictive representations that retain clearer links to biological meaning. Studies in toxicity prediction, protein-interaction modeling, and drug screening demonstrate the value of descriptor-based learning, but future models should be designed to identify which molecular or structural features are responsible for their predictions rather than only improving benchmark accuracy (Wu and Wei 2018; Wang et al. 2020; Feng et al. 2023). In single-cell and multimodal applications, progress will similarly depend on representations that remain stable across datasets, handle sparsity and missing modalities, and clarify the contribution of each data source (Fu et al. 2020; Liu et al. 2022; Yuan et al. 2025; Liang et al. 2026; Wang et al. 2025).

A third direction is the development of gray-box and inference methods in which learned components remain distinguishable from mechanistic parameters and retain a biologically meaningful interpretation. Universal differential equations, gray-box identification, and interpretable mechanism-discovery methods provide important starting points, but future work should place greater emphasis on constraints that prevent learned terms from absorbing arbitrary model error (El-Gazzar and Gerven 2025; Daryakenari et al. 2024; Massonis et al. 2023; VandenHeuvel et al. 2022). More reliable optimization methods will also be needed for difficult differential-equation settings, including singular perturbation problems (Chen et al. 2025).

For inference, future methods should combine structural information with adaptive simulation and experimental design. Rather than estimating parameters for a fixed simulator alone, these methods should help determine which observations are needed to distinguish competing mechanisms and when the assumed model should be revised (Durkan et al. 2018; Voznica et al. 2022; Wang et al. 2026; Raissi et al. 2019; Ning et al. 2023; Giampiccolo et al. 2024; Yazdani et al. 2020). This is particularly important for regulatory-network inference and single-cell trajectory reconstruction, where the scientific value of the result depends on whether the inferred structure can be tested and interpreted as a biological mechanism (Hossain et al. 2024; Hong et al. 2023; Ventre et al. 2023; Wang et al. 2019; Sha et al. 2024).

A fourth direction follows from the uncertainty taxonomy developed in Sect. 6. Future integrated models should propagate uncertainty from observations and biological dynamics through parameter estimation, latent-state reconstruction, learned model components, and intervention outcomes. In control, this means moving beyond policies optimized for one fitted model or nominal trajectory. The strongest existing examples already combine learning with mechanistic epidemic models, tumor models, and physiological feedback systems (Yin et al. 2023; Gallagher et al. 2024; Aiello et al. 2024; Böttcher et al. 2025). Future controllers should be robust to model discrepancy, account explicitly for competing objectives and safety constraints, and represent long-term treatment consequences (Zhang et al. 2026; Sharma et al. 2025; Laber et al. 2018; Liu et al. 2025; Chen et al. 2026).

Clinical decision problems also require methods that can learn from observational data without ignoring treatment-selection bias. Causal reinforcement learning, counterfactual policy transfer, and dynamic treatment regime methodology therefore remain important directions for connecting learned policies with clinically valid treatment comparisons (Tu et al. 2025; Zhang and Bareinboim 2020; Wu et al. 2024; Killian et al. 2022; Laber et al. 2014). Because relevant biological and clinical data are often distributed across institutions, federated and parameter-aware aggregation methods will also be needed to address cross-site heterogeneity without requiring the exchange of raw patient data (Chang et al. 2026).

A fifth, integrating direction is the development of mechanistic medical digital twins. Digital twins provide a natural framework for combining the four roles: inference updates latent states and personalized parameters, gray-box or mechanistic models propagate biological dynamics, prediction forecasts future outcomes, and control selects interventions. Within such a workflow, the distinct uncertainty sources identified in Sect. 6 should be propagated from patient measurements to forecasts and treatment decisions rather than collapsed into a single confidence estimate (Laubenbacher et al. 2022, 2024; Böttcher et al. 2025). Identifiability and observability are therefore central to whether a digital twin can be personalized and updated reliably.

As these directions develop, the boundaries among prediction, gray-box modeling, inference, and control will become less rigid. Surrogates may support inference and control, inferred structure may guide later intervention design, and mechanistically constrained learning may connect representation, simulation, and decision-making within a single workflow. The four-role taxonomy should therefore be applied to the function of each component rather than to an entire software platform or AI system. This preserves a clear basis for evaluation even when multiple methods are combined.

Overall, future progress is likely to come from coordinated frameworks that make AI more biologically grounded, dynamically explicit, uncertainty-aware, and useful for mechanistic reasoning and intervention, rather than from increasing model flexibility alone.

Realizing these opportunities will also require changes in training and community infrastructure. AI and machine learning should become part of the standard toolkit of mathematical biology, while researchers and students should have opportunities to learn how these methods can be integrated critically with mechanistic modeling. Tutorials, workshops, summer schools, conference sessions, open educational materials, and revisions to undergraduate and graduate curricula can help the community evaluate new methods and adopt them without losing the mathematical and biological foundations of the field.

## Conclusion

AI in mathematical biology is best understood not as a single methodological trend, but as a set of distinct roles defined by how it interacts with mechanistic models and biological reasoning. In this review, those roles are organized as prediction, hybrid modeling, inference, and control. Each serves a different scientific purpose, produces different kinds of outputs, and therefore requires different forms of evaluation and validation.

This role-based perspective helps clarify differences that are often hidden when the literature is organized only by disease area, data type, or algorithmic family. Similar AI tools can play different scientific roles, and their success should therefore be judged according to the claims they support. Predictive performance, dynamical fidelity, identifiability and calibrated uncertainty, or intervention safety may be central depending on whether the method is used for prediction, modeling, inference, or control.

Across these four roles, one central conclusion emerges: AI is most valuable in mathematical biology when it remains tied to biological structure rather than used as an isolated prediction engine. Whether the goal is to improve prediction, complete or accelerate mechanistic models, recover latent biological structure, or guide interventions, numerical success alone is insufficient; the method must also support reliable and interpretable biological understanding. Mechanistic digital twins illustrate this point especially clearly, since they require prediction, hybrid modeling, inference, and control to operate together within an uncertainty-aware and biologically interpretable workflow.

Ultimately, the future of AI in mathematical biology lies not in replacing mechanistic thinking, but in strengthening it by connecting predictive flexibility with biological structure, uncertainty-aware evaluation, and scientifically meaningful decision-making.

## Data Availability

No new datasets were generated or analyzed during the preparation of this review article.
